# Mobile phone applications to support psychotropic tapering: a scoping review protocol

**DOI:** 10.12688/hrbopenres.13501.2

**Published:** 2022-08-19

**Authors:** Miriam Boland, Agnes Higgins, Gavin Doherty, Greg Sheaf, Adele Framer, Cathal Cadogan

**Affiliations:** 1 School of Pharmacy and Pharmaceutical Sciences, Trinity College Dublin, Dublin, Ireland; 2 School of Nursing and Midwifery, Trinity College Dublin, Dublin, Ireland; 3 School of Computer Science and Statistics, Trinity College Dublin, Dublin, Ireland; 4 The Library of Trinity College, Trinity College Dublin, Dublin, Ireland; 5 SurvivingAntidepressants.org, San Francisco, California, USA

**Keywords:** Psychotropic, antidepressants, benzodiazepines, antipsychotics, withdrawal, tapering, discontinuation, smartphone apps, mental disorders, scoping review

## Abstract

**Background**: In the context of a recovery-oriented approach to mental healthcare, the role of psychotropic medication over extended or indefinite periods is increasingly being called into question. To minimise the risks of withdrawal symptoms and relapse, it is crucial that service users who want to discontinue psychotropic medication are supported throughout the tapering process. However, in the absence of effective interventions and supports, service users are increasingly relying on online resources for guidance and support. To date, the evidence base for mobile phone applications (‘apps’) and app-based interventions supporting discontinuation of psychotropic use has not been examined. This scoping review aims to examine the content, underpinning evidence base and impact of available mobile phone apps and app-based interventions to support psychotropic tapering.

**Methods** **:** A scoping review will be conducted using the Joanna Briggs Institute guidance and results will be reported using the Preferred Reporting Items for Systematic Reviews and Meta‐Analyses extension for Scoping Reviews (PRISMA-ScR) guideline. Several electronic databases (MEDLINE, EMBASE, CINAHL, PsycINFO, Web of Science, ACM and IEEE Xplore) will be searched from 2008 onwards. Searches of the major app stores will also be conducted, specifically Apple's App Store (iOS) and Google Play Store (Android). Following screening, key information will be extracted from the included studies and apps. Identified apps will be coded using the Behaviour Change Technique (BCT) Taxonomy. The findings will be described using narrative synthesis.

**Conclusions** **: **This scoping review will provide a broad overview of available apps to support psychotropic tapering, including a summary of their content using the BCT Taxonomy. The review findings will guide future research relating to the development, implementation and evaluation of app-based interventions to support the tapering of psychotropic medication.

## Introduction

Psychotropic medications, including antidepressants, anxiolytics, hypnotics, mood stabilisers and antipsychotics, are frequently prescribed for people experiencing mental health problems, with notable increases in prescribing rates observed year on year ^1, 2^. Globally, the total consumption of psychotropic medication increased by 4% annually from 2008 to 2019 ^3^. This surge in the use of psychotropic medication has contributed to the ongoing debate surrounding the over-prescribing of these medications and the potentially inappropriate treatment of mental health disorders. While guidelines published by the National Institute for Health and Care Excellence (NICE) support the use of psychotropic medication in the treatment of various mental disorders for a limited duration, evidence on long-term outcomes is lacking ^4, 5^.

The safety and efficacy of psychotropic medication is increasingly being debated, particularly in terms of the extent of any clinically meaningful improvements in symptoms when compared to placebo, and the potential for adverse effects, such as dependence and withdrawal symptoms ^6^. Studies have shown that 30–60% of long-term antidepressant prescriptions lack an indication for continued use and therefore may be inappropriate ^7, 8^. Similar concerns have been raised about antipsychotic and benzodiazepine use and their known potential risks. While evidence supports the use of antipsychotics in acute psychosis, evidence on their long-term use is lacking ^9^. This is acknowledged by other studies which estimate that approximately 40% of those taking antipsychotics would remain well (i.e. would achieve a good outcome in the long term) without them ^9^. With respect to benzodiazepine receptor agonists, while guidelines recommend that they should be limited to short‐term use (i.e., ≤4 weeks) to minimise the risk of adverse outcomes, long-term use of these medications is common ^10^. It is estimated that 3% of the general population use benzodiazepines for extended periods (>6 months) ^11^.

The role of psychotropic medication, particularly over extended or indefinite periods, is also being called into question with increasing advocacy of a recovery-oriented approach to mental healthcare. Despite the widespread use of psychotropic medication in treating mental health problems for many years, the mechanisms of action for drugs such as antidepressants and antipsychotics are still not fully understood. Previous theories that these drugs address an underlying chemical imbalance have found to be lacking any supporting evidence ^12, 13^. While recovery in mental health is difficult to define, personal recovery is about living a satisfying, meaningful and hopeful life despite the presence of challenges or the on-going limitations caused by mental health problems ^14– 16^. As such, a recovery-oriented approach is underpinned by values of service user engagement, respect for autonomy and personhood, including the right to be involved in decision making about care. Service users nationally and internationally have reported a high focus on medication adherence in their treatment, as well as a lack of collaboration in medication-related decisions, resulting in inability to make fully informed decisions with respect to their treatment ^17, 18^. This does not align with a rights-based, recovery-oriented approach to mental healthcare, which seeks to promotes open discussion about medication, including risks and benefits ^19^.

Psychotropic medications have various adverse effects. Examples include akathisia, involuntary movements, weight gain, and sexual dysfunction ^20^. Additionally, the use of psychotropic medication can lead to the development of physiological dependence with long-term use and withdrawal symptoms on discontinuation, which may ultimately play a role in extending their duration of use, even for those who have recovered during drug therapy or for whom there is no longer a valid indication to continue it ^21^. Some service users decide to discontinue psychotropic medication to recapture lost personal autonomy, to live a life free of medication and, in some cases, because these medications have failed to manage the person’s distressful experience and symptoms ^22^. However, these individuals have reported challenges in accessing professional support and having their autonomy and choice respected ^22, 23^.

Several studies have been conducted to investigate interventions that support the discontinuation of psychotropic medication and identify effective interventions that minimise the risk of relapse and withdrawal symptoms ^24– 26^. In many cases, the existing evidence base is limited. This can often be attributed to the lack of studies conducted over extended periods and the withdrawal confounding bias, i.e. misidentifying withdrawal symptoms as relapse ^20, 24^. A slow tapering method is often reportedly employed by individuals attempting psychotropic discontinuation and involves gradually reducing the dose of the psychotropic medication over a prolonged period of time ^27^. A conservative 10% dose reduction per month of the most recent dose is a common approach with some successes reported ^27, 28^. A similar approach involves switching to an alternative medication with a longer half-life before beginning gradual dosage reduction ^20, 29^.

The potential for withdrawal symptoms exists both during gradual reduction and following discontinuation of psychotropic medications. This can be explained by the neurophysiologic readjustments that take place in the body in the absence of the psychotropic agent ^30^. While the frequency and severity of withdrawal symptoms are difficult to ascertain, their rate of occurrence is potentially more common than reported due to the potential for misdiagnosis as an emergence or recurrence of a mental health problem ^31, 32^. While guidelines have previously suggested that withdrawal symptoms from psychotropic medications are limited to one to two weeks, this is increasingly contested, with evidence they may last for months ^33^. The list of withdrawal symptoms is extensive, with people reporting a range of symptoms (e.g. anxiety, tremors, lethargy) that range on a continuum of mild to severe ^32, 34^. Withdrawal symptoms can be debilitating and protracted, and are one of the main barriers reported by service users to successful discontinuation of psychotropic medication ^34^.

In the absence of effective interventions and supports, service users are increasingly relying on online resources, such as discussion fora and Facebook groups, for guidance and support while tapering psychotropic medicationt ^2, 27, 35^. In addition, the use and acceptability of mobile ‘apps’ defined as “ *a software program that runs on a mobile phone*” has been gaining momentum in healthcare ^36^. As of 2020, there were more than 10,000 mental health and wellness related apps available for download, which equates to one third of the app market ^37^. While the use and functioning of apps that promote adherence to psychotropic medication have been evaluated by several studies to date, the evidence base for apps and app-based interventions in supporting safe and effective discontinuation of psychotropic use has not been examined ^38^.

The aim of this scoping review is to examine the content, underpinning evidence base and impact of available mobile apps and app-based interventions to support psychotropic tapering. The objectives are to:

1.   Identify the available apps;

2.   Describe the apps in terms of functions, tapering recommendations and any linked services or supports to promote recovery;

3.   Examine the app development process (i.e. underpinning evidence and theory);

4.   Characterise the identified apps’ active components using the behaviour change technique (BCT) taxonomy ^39^; 

5.   Characterise the study populations included in evaluations of the apps;

6.   Identify the outcome measures that have been used to evaluate the apps;

7.   Summarise the impact of the identified apps based on the available evidence. 

## Methods

Given that the aim of this review is to provide a broad overview of the existing literature, a scoping review was deemed the more suitable review methodology, in comparison to a systematic review which tends to be more specific in nature. Scoping reviews explore the depth of existing literature, identify knowledge gaps and inform future research ^40^. The scoping review protocol presented in this manuscript has been developed in accordance with the Joanna Briggs Institute (JBI) guidance ^40, 41^. The Preferred Reporting Items for Systematic Reviews and Meta-Analyses guidelines for scoping reviews (PRISMA-ScR) will be used to guide the reporting of the final review ^42^. 

### Eligibility criteria

The Population-Intervention-Comparison-Outcome (PICO) framework will be used to guide study selection and to align the eligibility criteria with the aims of this review.

***Types of participants.*** This review will include apps and app-based interventions targeting individuals who want to discontinue psychotropic medication use. For the purpose of this review, studies and apps that include or target adults (≥18 years) taking any form of psychotropic medication will be included (e.g. antidepressants, anxiolytics, antipsychotics and/or mood stabilisers).

***Types of interventions.*** Eligible apps and app-based interventions must focus on supporting the process of psychotropic tapering. Tapering-related guidance can come from any source. Eligible apps will include those available from publicly available commercial apps, as well as those identified through electronic database searching. Apps and interventions which only focus on medication adherence will be excluded.

***Types of outcome measures.*** Given the lack of existing reviews evaluating app-based interventions to support psychotropic tapering, all outcomes for studies that meet the pre-specified inclusion criteria will be included in this review. These will include quantitative outcome measures and qualitative feedback reported by service users and/or service providers, as well as proximal outcomes such as engagement and usage. This will enable a comprehensive overview of outcome measures to be generated.

***Types of studies.*** All studies evaluating apps and app-based interventions meeting the above criteria will be included in this review. This will include quantitative evaluations (e.g. randomised controlled trials, before and after studies) and qualitative evaluations (e.g. interview studies). Apps identified solely through online stores that have not undergone any formal evaluation will also be eligible for inclusion. Only studies and apps published in the English language will be considered for inclusion.

### Search strategy

The search strategy will consist of two components: (1) a search of electronic databases to identify empirical research; and (2) a search of mobile phone application stores (‘app stores’) to identify commercially available apps ^43^.

Component one will include a search of the following electronic databases and digital libraries: MEDLINE, EMBASE, CINAHL, PsycINFO, Web of Science, ACM and IEEE Xplore. All databases and libraries will be searched from 2008, the year the first app store (Apple) was introduced. The electronic database searches will consist of three steps:

1. A preliminary search of one electronic database (Web of Science) will be undertaken to identify articles relevant to the review topic. This will enable the identification of keywords and index terms from the titles and abstracts of relevant articles.
2. Based on the above, a comprehensive search strategy will be designed and refined in collaboration with a research/medical librarian (GS), with expertise in information retrieval and systematic reviews.
3. A search of the databases listed above will be conducted using all identified keywords and index terms, modified to each database accordingly.

Component two will include a search of the following app stores from their inception: Apple's App Store (iOS) and Google Play Store (Android). This will involve:

1.   A preliminary search of Apple’s app store to identify keywords and apps relevant to the review topic. This initial search will enable the identification of the names and descriptions of relevant apps and guide future searches.

2.   Informed by these findings, a comprehensive search strategy will be designed and refined in conjunction with a co-author with expertise in app searching (GD).

3.   A systematic search of the two platforms will be conducted using the identified keywords. Separate searches for each keyword will be conducted on each platform, across all categories of apps. Search results will be downloaded and stored for screening. 

Initial search terms for online peer-reviewed papers will include: antidepressants, benzodiazepines, anxiolytics, taper, psychotropic, withdrawal, discontinuation, depression, anxiety, psychosis, mobile phone applications, apps. The complete list of search terms will be included in the final review manuscript. Reference lists of included studies will be screened for additional studies.

### Study selection

Following completion of the electronic database searches, identified references will be imported into Covidence (reference management software platform; https://www.covidence.org/) and undergo deduplication. A two-step screening process will be used:

1. Two researchers (MB, CC) will independently screen titles and abstracts for inclusion based on the pre-specified inclusion criteria outlined above.
2. If a study appears to meet inclusion criteria or a decision cannot be made based on the title and abstract alone, the full-text article will be retrieved. All full-text articles will be screened independently by both researchers. Any disagreements throughout the screening process will be resolved by consensus with the input of a third reviewer (AH) where required.

A similar process will be undertaken with the app store searches. Details of all potentially relevant apps identified from the app store searches will be entered into Microsoft Excel by the lead researcher (MB). The following details will be recorded for each app: the name, brief description of its functions, the app store and app store category, age category, author/creator and a link to access the app. All apps will be screened for inclusion by two researchers independently (MB, CC). The third reviewer (AH) will again be consulted in the event of any disagreements.

The study selection process will be described both narratively and using a PRISMA-ScR flow diagram, detailing all the steps involved. A list of excluded apps and articles will be provided as an appendix which will include reasons for exclusion.

### Data extraction

Two researchers (MB, CC) will independently perform data extraction (‘data charting’) using a data extraction form/charting table that will be developed in Microsoft Excel. This will be developed in accordance with the relevant methodological guidance to record key information from each source. The data extraction form will be piloted on one study and one app meeting inclusion criteria and refined accordingly. 

Key information to be extracted will include:

1. Study/App: author(s)/creator(s), year of publication/creation, origin/country of origin, aims/purpose;
2. Target population;
3. Intervention/App content (including BCTs, app functions and linked services/intervention);
4. App development process to ascertain quality (including details of any underpinning clinical evidence, theory and/or evidence of human-centred design process);
5. Study design, sample size and outcome measures (if available);
6. Outputs and reports;
7. Key findings of the evaluations of any identified apps.

### Data analysis and synthesis

The JBI guidance recommends that the analysis of data be pre-specified within the protocol to ensure transparency and justification ^40, 41^. Given that the aim of this scoping review is to provide a broad overview of the existing literature and commercially available apps and not to pool outcome data, narrative synthesis will be used to describe the key findings and how they relate to the review’s objectives. The approach to narrative synthesis will be informed by the Synthesis Without Meta-analysis (SWiM) guidelines ^44^. This will help to address the common issues that have been identified with narrative synthesis, including a lack of description of the methods used and inadequate reporting of the limitations of the synthesis. Key study and app information will be summarised and then presented in tabular form. This will be accompanied by a narrative summary which will describe how these results relate to the review objectives and questions. This process will be further developed and refined throughout the data extraction process.

### App coding

Identified apps and app-based interventions will be coded using the Behaviour Change Technique Taxonomy version 1 (BCTTv1) ^39^. A BCT is defined as *“observable, replicable, and irreducible component of an intervention designed to alter or redirect causal processes that regulate behaviour”* ^39^. The BCT Taxonomy consists of 93 BCTs, grouped within 16 categories with detailed definitions of each BCT ^39^. The application of this taxonomy will enable the identification and characterisation of the identified apps’ active components using standardised terminology ^25^. This will ultimately allow for greater comparison and replication of any identified interventions ^45^.

Apps will be downloaded from the app stores by two researchers (MB, CC). This will enable the functionality of these apps to be assessed. To account for the potential of longitudinal features offered by apps, the researchers will set up a profile on each app using a sample patient case (e.g. tapering from 10 mg diazepam three times daily). This will enable them to fully explore the apps’ features and functionality, including any notifications and usage metrics over a fixed period of time (e.g. four weeks). Screenshots of the content and outputs of each app will be retained to facilitate app coding. The app coding process will consist of three stages.

1. Preliminary coding of a random sample of 20% of identified apps will be undertaken by two researchers (MB, CC) using the entire BCT taxonomy.
2. A coding manual will be developed which will include definitions for the subset of identified BCTs and examples of how they apply in the context of the identified app/interventions.
3. BCT coding of the apps/interventions will be conducted by two researchers (MB, CC) independently using the coding manual, which will be refined on an iterative basis.

Any disagreements will be resolved by consensus or through discussion with a third reviewer (AH).

### Critical appraisal

Given that the aim of this scoping review is to provide a broad overview of the existing literature and apps and not to pool outcome data ^40^, the critical appraisal process will focus on the potential for bias and the extent to which it has been addressed by the included studies. Critical appraisal of included studies will be performed by two researchers (MB, CC) working independently using the relevant JBI Critical Appraisal Checklist for different study types ^46^. The critical appraisal process will not be used to exclude studies, but to help inform the synthesis and interpretation of the review findings.

### Presentation of results

The JBI guidance recommends that scoping review protocols include a proposed plan for presenting results. This plan will be further refined at the review stage based on the identified studies and the charted data. The results will be collated and summarised according to the review objectives and eligibility criteria (PICO framework). Key findings will be presented under the following headings and using a PRISMA-ScR flow diagram:

1. Search results
2. Aims/purpose of identified apps
3. Target population
4. Intervention/App content (including BCTs, app functions and linked services/intervention)
5. App development process
6. Study design, sample size and outcome measures
7. Key findings

## Discussion

The use of psychotropic medications is becoming more widespread, with about a third of service users receiving long-term treatment despite insufficient medical indication ^8^. Although potential benefits are associated with the use of psychotropic medications in certain circumstances, they also have various associated adverse effects. Increasingly, it is recognised that psychotropic medication may not be needed or appropriate over extended periods, and service users are seeking support in safely discontinuing treatment.

This review will identify apps and app-based interventions to support the discontinuation of psychotropic medications. It will provide a broad overview of available apps, including a summary of their content using the BCT Taxonomy. The review will also provide a summary of existing evaluations of the effectiveness of these app. The review findings will guide future research relating to the development, implementation and evaluation of app-based interventions to support the tapering of psychotropic medications.

### Dissemination of findings

The completed review will be submitted for publication in a peer-reviewed journal.

### Study status

At the time of publication of this protocol, database searches have yet to be completed.

## Data availability

### Underlying data

No underlying data are associated with this article.

### Extended data

Open Science Framework: A scoping review of mobile phone applications to support psychotropic tapering (protocol). https://doi.org/10.17605/OSF.IO/ZGBKF ^43^

This project contains the following extended data:

- WebOfScience_Strategy_OSF.docx (Web of Science Search Strategy)

### Reporting guidelines

Open Science Framework: PRISMA-P checklist for ‘Mobile phone applications to support psychotropic tapering: a scoping review protocol’. https://doi.org/10.17605/OSF.IO/ZGBKF ^43^

Data are available under the terms of the Creative Commons Zero "No rights reserved" data waiver (CC0 1.0 Public domain dedication).
