# Supplementary material for: Mobile phone applications to support psychotropic tapering: a scoping review protocol
Source: HRB Open Res. 2022 Aug 19;5:18. Originally published 2022 Mar 8. [Version 2] doi: 10.12688/hrbopenres.13501.2 (PMC9532954; doi:10.12688/hrbopenres.13501.2)
Supplement: Peer review history

# Peer review: Reviewer response for version 2

**DOI:** 10.21956/hrbopenres.14874.r32714

**Published:** 2022-10-04

**Authors:** Mark Horowitz

**Affiliations:** 1 Division of Psychiatry, University College London, London, UK

Thank you for incorporating my feedback into your protocol and for responding to my concerns.

Is the study design appropriate for the research question?

Partly

Is the rationale for, and objectives of, the study clearly described?

Yes

Are sufficient details of the methods provided to allow replication by others?

Yes

Are the datasets clearly presented in a useable and accessible format?

Not applicable

Reviewer Expertise:

I study rationale psychopharmacology and patient-centred deprescribing of psychotropic drugs

I confirm that I have read this submission and believe that I have an appropriate level of expertise to confirm that it is of an acceptable scientific standard.

# Peer review: Reviewer response for version 1

**DOI:** 10.21956/hrbopenres.14726.r32384

**Published:** 2022-07-29

**Authors:** Luke Molloy

**Affiliations:** 1 School of Nursing, University of Wollongong, Wollongong, NSW, Australia

There is a clear rationale for this proposed review. The area is of interest to mental health-related clinicians and academics, and I am sure there will be interest in its findings across disciplines. There is a clear narrative and excellent use of literature within the sections establishing this. A scoping review is established as the most appropriate approach to understanding this phenomenon, and the details of the methods are clear and would allow replication of the work to be undertaken. The findings of such a work will support quality recovery-focused mental health care.

Is the study design appropriate for the research question?

Yes

Is the rationale for, and objectives of, the study clearly described?

Yes

Are sufficient details of the methods provided to allow replication by others?

Yes

Are the datasets clearly presented in a useable and accessible format?

Yes

Reviewer Expertise:

Mental health care, trauma-informed care, strengths-based practice.

I confirm that I have read this submission and believe that I have an appropriate level of expertise to confirm that it is of an acceptable scientific standard.

# Response

**Published:** 2022-08-16

**Authors:** Miriam Boland

**Affiliations:** University of Dublin, Ireland

There is a clear rationale for this proposed review. The area is of interest to mental health-related clinicians and academics, and I am sure there will be interest in its findings across disciplines. There is a clear narrative and excellent use of literature within the sections establishing this. A scoping review is established as the most appropriate approach to understanding this phenomenon, and the details of the methods are clear and would allow replication of the work to be undertaken. The findings of such a work will support quality recovery-focused mental health care.

**Response:** We are very grateful to the reviewer for reviewing our manuscript and for the positive feedback they have provided.

# Peer review: Reviewer response for version 1

**DOI:** 10.21956/hrbopenres.14726.r31734

**Published:** 2022-04-04

**Authors:** Mark Horowitz

**Affiliations:** 1 Division of Psychiatry, University College London, London, UK

Thank you for giving me the opportunity to review this timely proposed review. This study will produce a useful overview of existing mobile apps for the process of deprescribing psychiatric medication, a topic which has been until recently ignored, and this review is an important step forward, given how ubiquitous apps are for every facet of human life – and I congratulate the authors for taking on this project in this important area.

 Having said that there are limitations which should be addressed to make this study as useful as possible for researchers in this area. My major issue relates to the method by which the apps are evaluated.

 The major rationale to evaluate apps is to see which are most effective – given that a trial of the apps is not proposed, the next best criteria would be to assess which apps adhere most closely to accepted ‘best practice’ for tapering or which generate the greatest user satisfaction. I can see that outcomes if measured will be included in appraisal under ‘key findings’.

 I am not sure that assessing the development process of an app allows this to be established. An app could be established through a very comprehensive process and still fail to generate an app that is useful to patients.

 Therefore I wonder whether an additional frame is used to evaluate the apps: namely, fidelity to best practice. Best practice is itself a quandary, in this under-studied area, but I would suggest RCPsych’s guidance on stopping antidepressants, or an approach as outlined in Framer (2021 ^1^), with NICE guidance for the other relevant classes also applicable.

 Perhaps, similarly to this appraisal of existing CPGs for deprescribing, some domains of good practice could be established, against which existing apps are evaluated: Sørensen *et al. *(2022 ^2^).

 Additionally, I see a major problem is with the approach of coding using the BCT taxonomy. In the introduction the authors state that the main cause of difficulty stopping these medications is physiological dependence that leads to withdrawal on stopping.

 A BCT taxonomy seems to be a framework for changing behaviour. This seems to imply its relevance is most geared to modifying addictive behaviour – for example, stopping compulsive smoking. However with physiological dependence there is not a learned behaviour that needs to be modified – antidepressant, and antipsychotic users are not using these drugs compulsively or in an addictive manner, and this is also true for the majority of users of benzos and z-drugs. People who want to stop these medications mostly would like to throw them in the bin. But they are unable to do so because of the aversive physiological consequences that result.

 It therefore does not make sense to me to apply a BCT approach to these apps that are trying to achieve something quite different. From my knowledge of these apps, they attempt to help patients make calculations regarding hyperbolic or exponential dose reductions, log withdrawal symptoms and provide motivation and reassurance through a physiologically unpleasant trajectory.

 I fear applying a BCT lens to this implies that difficulty stopping comes down to a learnt behaviour i.e. a predominantly psychological process and that in doing so it will miss the main ingredients.

 Perhaps a less rigid approach that does not bring to bear the assumptions of a BCT frame would be best, perhaps adapted from this framework if the authors believe there are elements that are useful?

 How will apps that were not developed through academic processes (the vast majority of them presumably) be evaluated? Mention is made of trialling with a user – will their satisfaction be measured? Is pretending to be someone on 10mg TDS of diazepam really likely to generate a useful output of what using the app is like? It may be better to either say you will simply list the elements of these apps or involve someone actually tapering to trial these apps (which might be a more involved and different study)? Will the developer of these apps be written to in order to inquire about their development process?

Minor points:

 Intro:

 Psychotropic medication has increased by 4% annually – over what time period?

 There are several analyses which find that the difference between placebo and antidepressant is not clinically significant (though it is statistically significant), leading to queries from Cochrane reviews, and others about whether these drugs should be used at all. This should inform this introduction.

 It is also worth pointing out that the neurotransmitter deficiencies that are often cited as rationales for medication in mental health conditions are now generally rejected and so a biological rationale for the treatment of distressing experiences is not solid. A ‘recovery’ approach in this frame may represent simply a common sense, reality-based approach to care.

 Discussion of BZRAs – this statement regarding the evidence for benefit in long-term use is lacking is not accurate: most guidelines recommend that they are not used for more than 2-4 weeks and people using them longer than this should stop them. Their harms and benefits are similar to benzos to which they are almost identical. This section should be less equivocal.

 Each of the domains of withdrawal symptoms mentioned on p.3 could be mild, moderate and severe – there are mild tremors and severe anxiety (panic attacks) so please re-phrase this section.

Is the study design appropriate for the research question?

Partly

Is the rationale for, and objectives of, the study clearly described?

Yes

Are sufficient details of the methods provided to allow replication by others?

Yes

Are the datasets clearly presented in a useable and accessible format?

Not applicable

Reviewer Expertise:

I study rationale psychopharmacology and patient-centred deprescribing of psychotropic drugs

I confirm that I have read this submission and believe that I have an appropriate level of expertise to confirm that it is of an acceptable scientific standard, however I have significant reservations, as outlined above.

## References

[1] : What I have learnt from helping thousands of people taper off antidepressants and other psychotropic medications. *Ther Adv Psychopharmacol* .2021;11: 10.1177/2045125321991274 2045125321991274 10.1177/2045125321991274 33796265PMC7970174

[2] : Clinical practice guideline recommendations on tapering and discontinuing antidepressants for depression: a systematic review. *Ther Adv Psychopharmacol* .2022;12: 10.1177/20451253211067656 20451253211067656 10.1177/20451253211067656 35173954PMC8841913

# Response

**Published:** 2022-08-16

**Authors:** Miriam Boland

**Affiliations:** University of Dublin, Ireland

Thank you for giving me the opportunity to review this timely proposed review. This study will produce a useful overview of existing mobile apps for the process of deprescribing psychiatric medication, a topic which has been until recently ignored, and this review is an important step forward, given how ubiquitous apps are for every facet of human life – and I congratulate the authors for taking on this project in this important area.

**Response**: We are very grateful to the reviewer for reviewing our manuscript and for the feedback they have provided. We have responded to each comment below and revised the manuscript accordingly. We have indicated the page numbers within the revised manuscript where revisions have been made (all visible as tracked changes).

 1. Psychotropic medication has increased by 4% annually – over what time period?

**Response**: We have now clarified the time period in the introduction.

*“Globally, the total consumption of psychotropic medication increased by 4% annually from 2008 to 2019 (3).”* (Page 3)

 2. There are several analyses which find that the difference between placebo and antidepressant is not clinically significant (though it is statistically significant), leading to queries from Cochrane reviews, and others about whether these drugs should be used at all. This should inform this introduction.

**Response**: We have amended this part in the introduction section to address this point. However, we do not wish to weigh in on the ongoing debate about whether antidepressants should be used as this is beyond the scope of this review:

*“The safety and efficacy of psychotropic medication is increasingly being debated, particularly in terms of the extent of any clinically meaningful improvements in symptoms when compared to placebo, and the potential for adverse effects, such as dependence and withdrawal symptoms (6).* *Studies have shown that 30-60% of long-term antidepressant prescriptions lack an indication for continued use and therefore may be inappropriate (7, 8).* *Similar concerns have been raised about antipsychotic and benzodiazepine use and their known potential risks. While evidence supports the use of antipsychotics in acute psychosis, evidence on their long-term use is lacking (9). This is acknowledged by studies which estimate that approximately 40% of those taking antipsychotics would remain well (i.e., would achieve a good outcome in the long term) without them (9).” *(Page 3)

 3. It is also worth pointing out that the neurotransmitter deficiencies that are often cited as rationales for medication in mental health conditions are now generally rejected and so a biological rationale for the treatment of distressing experiences is not solid. A ‘recovery’ approach in this frame may simply represent a common sense, reality-based approach to care.

**Response:** We have amended this section, so that it reads as follows:

*“Despite the widespread use of psychotropic medication in treating mental health problems for many years, the mechanisms of action for drugs such as antidepressants and antipsychotics are still not fully understood. Previous theories that these drugs address an underlying chemical imbalance have found to be lacking any supporting evidence (12,13).”* (Page 3)

 4. Discussion of BZRAs – this statement regarding the evidence for benefit in long-term use is lacking is not accurate: most guidelines recommend that they are not used for more than 2-4 weeks and people using them longer than this should stop them. Their harms and benefits are similar to benzos to which they are almost identical. This section should be less equivocal.

**Response:** We have reworded this sentence as follows:

*“With respect to benzodiazepine receptor agonists, while guidelines recommend that they should be limited to short‐term use (i.e., ≤4 weeks) to minimise the risk of adverse outcomes, long-term use of these medications is common. It is estimated that 3% of the general population use benzodiazepines for extended periods (>6 months) (10,11).”*(Page 3)

 5. Each of the domains of withdrawal symptoms mentioned on p.3 could be mild, moderate and severe – there are mild tremors and severe anxiety (panic attacks) so please re-phrase this section.

**Response:** We have reworded this sentence as follows:

*“The list of withdrawal symptoms is extensive, with people reporting a range of symptoms (e.g., anxiety, tremors, lethargy) that range on a continuum of mild to severe (32, 34).” *(Page 5)

 6. The major rationale to evaluate apps is to see which are most effective – given that a trial of the apps is not proposed, the next best criteria would be to assess which apps adhere most closely to accepted ‘best practice’ for tapering or which generate the greatest user satisfaction. I can see that outcomes if measured will be included in appraisal under ‘key findings’.

 I am not sure that assessing the development process of an app allows this to be established. An app could be established through a very comprehensive process and still fail to generate an app that is useful to patients. Therefore I wonder whether an additional frame is used to evaluate the apps: namely, fidelity to best practice. Best practice is itself a quandary, in this under-studied area, but I would suggest RCPsych’s guidance on stopping antidepressants, or an approach as outlined in Framer (2021), with NICE guidance for the other relevant classes also applicable.

 Perhaps, similarly to this appraisal of existing CPGs for deprescribing, some domains of good practice could be established, against which existing apps are evaluated: Sørensen et al. (2022).

**Response:** The purpose of this scoping review is to provide a broad overview of the existing literature on apps, as well as commercially available apps. We are interested in looking at the apps in terms of their content, as well as their development, evaluation and reporting. Equally, if we establish that there are deficits in terms of how apps/studies are developed, evaluated and reported, that will provide useful information to inform future research.

 While we will be looking to describe the tapering recommendations and establish which sources of evidence, if any, the apps report as the basis for instructions on tapering, we have purposefully avoided selecting any single source as a gold standard to critique the recommendations as we realise that there is no universally accepted guidance on tapering.  However, we do plan on putting the findings of the review on this topic in context and discussing different sources of guidance on tapering in the discussion section of the completed review. 

 7. Additionally, I see a major problem is with the approach of coding using the BCT taxonomy. In the introduction the authors state that the main cause of difficulty stopping these medications is physiological dependence that leads to withdrawal on stopping.

 A BCT taxonomy seems to be a framework for changing behaviour. This seems to imply its relevance is most geared to modifying addictive behaviour – for example, stopping compulsive smoking. However with physiological dependence there is not a learned behaviour that needs to be modified – antidepressant, and antipsychotic users are not using these drugs compulsively or in an addictive manner, and this is also true for the majority of users of benzos and z-drugs. People who want to stop these medications mostly would like to throw them in the bin. But they are unable to do so because of the aversive physiological consequences that result.

 It therefore does not make sense to me to apply a BCT approach to these apps that are trying to achieve something quite different. From my knowledge of these apps, they attempt to help patients make calculations regarding hyperbolic or exponential dose reductions, log withdrawal symptoms and provide motivation and reassurance through a physiologically unpleasant trajectory.

 I fear applying a BCT lens to this implies that difficulty stopping comes down to a learnt behaviour i.e. a predominantly psychological process and that in doing so it will miss the main ingredients.

 Perhaps a less rigid approach that does not bring to bear the assumptions of a BCT frame would be best, perhaps adapted from this framework if the authors believe there are elements that are useful?

**Response**: For this review, the core focus of the apps that we are interested in looking at is the tapering of psychotropic medication. Even if dependence itself is not a learned behaviour, identifying solutions for helping people to taper and framing them in terms of BCTs could help ensure that they are described using a common terminology. Applying the BCT taxonomy allows for the content of any intervention to be defined using standardised terminology which will aid both the comparison of any identified interventions, as well as potential for future replication.

 As per the nature of a scoping review which is intended to be broad and exploratory, if we identify important features of any apps that cannot be coded using this taxonomy, then we will report that. This could ultimately help to stimulate future research on how best to describe the content of tapering apps using standardised terminology.

 8. How will apps that were not developed through academic processes (the vast majority of them presumably) be evaluated? Mention is made of trialling with a user – will their satisfaction be measured? Is pretending to be someone on 10mg TDS of diazepam really likely to generate a useful output of what using the app is like? It may be better to either say you will simply list the elements of these apps or involve someone actually tapering to trial these apps (which might be a more involved and different study)? Will the developer of these apps be written to in order to inquire about their development process?

**Response:** The evaluation of apps that are available on the app stores will primarily focus on the app content and functions. Once these apps have been identified, we will attempt to contact the app developers via email to ask for more information on the app development process.

 The purpose of trialling the apps with a sample user profile is not to assess the user satisfaction (this would require an actual research study with a sample of participants to gather quantitative and/or qualitative data), but to enable us as the reviewers to gain a better understanding of the features and functions offered by the apps.
